# Glatiramer acetate desensitisation in multiple sclerosis patients - the case report

**DOI:** 10.1186/2045-7022-4-S3-P57

**Published:** 2014-07-18

**Authors:** Bronislava Novotna, Jan Trna, Libuse Prochazkova, Hana Smerkova, Petra Praksova

**Affiliations:** 1 University Hospital Brno, Allergology, Dept of Internal Medicine, Czech Republic; 2 University Hospital Brno, Dept of Internal Medicine and Gastroenterology, Czech Republic; 3 University Hospital Brno, Hospital Pharmacy, Czech Republic; 4 Viameda s.r.o., Immunology Laboratory, Czech Republic; 5 University Hospital Brno, Department of Neurology, Czech Republic

## Background

Glatiramer acetate (GA) is immunomodulatory drug widely prescribed for the treatment of multiple sclerosis (MS). GA treatment is frequently associated with local site reactions and generalized urticaria. The systemic reaction approximately occurs in 10% of patients. The desensitization could help to return GA to MS treatment.

## Objective

Our aim was to evaluate the safety and efficiency of GA desensitisation. Eight days of the GA treatment MS female patient came with adverse reactions. Twelve hours after GA administration itching in the injection site, feeling of swelling tongue, swallowing difficulty and cough appeared.

## Methods

The 38-year-old woman with a diagnosis of MS was examined from April until July 2013 at the Allergology Unit, Department of Internal Medicine and Gastroenterology. In the absence of specific IgE tests against GA, a basophil activation test (BAT) was performed with a borderline result (GA concentration 1.0 ul/ml with 7.8% activated basophils, stimulation index 2.1). Then followed the GA skin tests with a positive results (skin prick test was negative, i.d. dilution 1:100 and 1:10 were positive (6 and 7.6mm weal diameter). Finally, on 11th July, 2013, after written informed patient consent, a 4-hour outpatient GA desensitisation procedure was carried out. Beginning with 20ng, we administered subcutaneous GA suspension in increasing dosage every 30 minutes to the target dose of 20 mg GA. Patients outcomes were monitored by return visit and by telephone follow-up.

## Results

No episodes of anaphylaxis or other adverse reaction occurred during or immediately after desensitisation. Our patient was able to successfully continue GA therapy (20 mg GA s.c. daily).

## Conclusions

In MS patients with adverse local or systemic reactions to GA, desensitization can be a solution allowing the return of GA treatment and improve the course of MS disease.

## Competing interests

The authors declare no conflict of interest.

